# Remote High-Definition Rotating Video Enables Fast Spatial Survey of Marine Underwater Macrofauna and Habitats

**DOI:** 10.1371/journal.pone.0030536

**Published:** 2012-02-27

**Authors:** Dominique Pelletier, Kévin Leleu, Delphine Mallet, Gérard Mou-Tham, Gilles Hervé, Matthieu Boureau, Nicolas Guilpart

**Affiliations:** 1 Unité de Recherche Lagons, Ecosystèmes et Aquaculture Durable en Nouvelle-Calédonie, French Institute for the Exploitation of the Sea, Nouméa, New Caledonia; 2 Unité de Recherche CoReUs, Institut de Recherche pour le Développement, Nouméa, New Caledonia; 3 Laboratoire de Biologie Halieutique, French Institute for the Exploitation of the Sea, Plouzané, France; 4 Laboratoire Environnement et Ressources PACA, French Institute for the Exploitation of the Sea, La Seyne-sur-Mer, France; University of Canterbury, New Zealand

## Abstract

Observing spatial and temporal variations of marine biodiversity from non-destructive techniques is central for understanding ecosystem resilience, and for monitoring and assessing conservation strategies, e.g. Marine Protected Areas. Observations are generally obtained through Underwater Visual Censuses (UVC) conducted by divers. The problems inherent to the presence of divers have been discussed in several papers. Video techniques are increasingly used for observing underwater macrofauna and habitat. Most video techniques that do not need the presence of a diver use baited remote systems. In this paper, we present an original video technique which relies on a remote unbaited rotating remote system including a high definition camera. The system is set on the sea floor to record images. These are then analysed at the office to quantify biotic and abiotic sea bottom cover, and to identify and count fish species and other species like marine turtles. The technique was extensively tested in a highly diversified coral reef ecosystem in the South Lagoon of New Caledonia, based on a protocol covering both protected and unprotected areas in major lagoon habitats. The technique enabled to detect and identify a large number of species, and in particular fished species, which were not disturbed by the system. Habitat could easily be investigated through the images. A large number of observations could be carried out per day at sea. This study showed the strong potential of this non obtrusive technique for observing both macrofauna and habitat. It offers a unique spatial coverage and can be implemented at sea at a reasonable cost by non-expert staff. As such, this technique is particularly interesting for investigating and monitoring coastal biodiversity in the light of current conservation challenges and increasing monitoring needs.

## Introduction

Conserving marine biodiversity is a global concern exemplified by the programme of work of the Convention on Biological Diversity (CBD) to “promote political actions for addressing biodiversity loss and the degradation of ecosystems and ecosystem services, as well as their implications for human well-being” (www.cbd.int/doc/decisions/cop-10/cop-10-dec-11-en.pdf). Marine Protected Areas (MPAs) are a key instrument for the conservation of marine biodiversity and associated ecosystem services [1]. Within the CDB, the quantitative targets for a global coherent network of MPAs set in 2002 were updated in 2010, with requirements for grounding the design of MPAs in the best available scientific knowledge, and to assess the performance of these MPAs to achieve their conservation objectives. Along with the implementation of conservation and restoration strategies for biodiversity, CDB urges Parties “to promote the generation and use of scientific information, develop methodologies and initiatives to monitor status and trends of biodiversity and ecosystem services, share data, develop indicators and measures, and undertake regular and timely assessments” [2].

However, in many areas around the world, the state and evolution of marine biodiversity remains unknown or poorly evaluated. This is primarily due to the lack of comprehensive and comparable field data, in relation with insufficient human and financial resources. The scarcity of data hampers the study of spatial and temporal patterns and variations in biodiversity facing stressors such as anthropogenic pressures and environmental changes. Appraising and understanding these variations is nevertheless indispensable for the understanding of ecosystem resilience, and such observations are central to the design, monitoring and assessment of biodiversity conservation strategies, including e.g. MPA.

Devising cost-effective and non-destructive observation techniques that permit collecting data with an appropriate spatial and temporal coverage is thus a timely challenge. With respect to underwater macrofauna and habitat, Underwater Visual Censuses (UVC) realized by divers have been widely used for monitoring coral reefs and temperate coastal ecosystems, and in particular macrofauna and benthic cover, e.g. [3]. Advantages and disadvantages of UVC for estimating fish abundance and diversity have been reported and discussed in many papers, among others [4], [5], [6]. The presence and abundance of vagile species at the observation location is significantly affected by the presence of a diver underwater [7], [8] and some species may not be well observed [9]. Furthermore, the level of experience of the diver is a source of heterogeneity in the data [10], and estimations of both fish size and distance are subject to uncertainties [11], [12]. A recent study quantified the consequences of such diver effects upon the abundance of reef fish groups [13].

In terms of capacity, UVC require experienced divers that are able to identify species and estimate individual fish sizes. Other drawbacks of UVC lie in the reduced number of observations that can be achieved within a given day, the limited depth range and the logistics of scuba diving, resulting in rather high field costs. For this reason, UVC are not conducted systematically in every habitat. Most often, the habitats where species are the most abundant, e.g. reef habitats, are preferred, whilst observations in soft-sediment areas are scarce.

Aside from UVC, video techniques have been increasingly used for observing underwater macrofauna and habitat, particularly in the last decade [14], [15]. Most video techniques that do not need the presence of a diver use baited remote systems (Baited Remote Underwater Video, BRUV) [16], [17]. BRUV attract a range of species beyond the carnivorous ones, and have also been used for studying fish assemblages, particularly using stereo-video [18], [19], [20]. The latter was developed to improve the estimation of fish size and distance [11]. A drawback of baited video lies in bait attraction which selectively influences species, thereby affecting the assessment of fish community structure [21], and the bait plume is difficult to evaluate [22]. From a technical standpoint, baiting requires to leave the system in place long enough to ensure bait effectiveness, and the vertical recording of images is not suited for observing habitat around the station.

Understanding biodiversity patterns and their evolution within an ecosystem approach to management (http://www.unep.org/ecosystemmanagement/) requires comparable observations in all habitats. This is also indispensable for monitoring the response of biodiversity to conservation strategies. Techniques that can be deployed in all habitats, and do not require divers nor expert staff on the field are preferred by environmental offices and managers.

This paper focuses on the need to devise observation techniques that offer a good spatial coverage and a holistic approach of macrobiodiversity, in the light of current conservation challenges and increasing monitoring needs. We present an original video technique (STAVIRO-***STA*** *tion* ***VI*** *deo* ***RO*** *tative in French*) that was developed and tested in a highly diversified coral reef ecosystem. The technique utilizes a High Definition (HD) camera enclosed in a rotating unbaited system, which was designed to ensure a minimum disturbance of species. Recorded images are analysed at the office to quantify biotic and abiotic sea bottom cover, and to identify and count fish species and some other species such as marine turtles, marine mammals and snakes. The technique was tested in the South Lagoon of New Caledonia in 2007, and then used each year from 2008 to 2010. Video transects using the same camera were carried out in 2007 and directly compared with UVC (Pelletier et al. 2011).

We reported here the findings of the 2007 survey for both macrofauna and habitat. First, we investigated the range of macrofauna species and abundances observed and identified from the images. Secondly, habitat data were analyzed to derive a typology of stations. In a third step, a range of biodiversity metrics pertaining to several components of macrofauna were computed from the data and their spatial variations were analysed in relation with protection status and habitat. Finally we discussed the advantages, shortcomings and complementarity of STAVIRO and UVC techniques for observing coastal biodiversity.

## Materials and Methods

### Ethics Statement

No specific permits were required for the described field studies. During the field study, only the video systems were immersed in water; no animals were collected or manipulated. This activity did not require any permission in the study area, and fully complied with New Caledonian environmental regulations (Code of the Environment, http://www.province-sud.nc/images/stories/pdf/environnement/Code.pdf).

### Study area

The study area (22°22.5°S, 166°14′E) was located in the Southwest Lagoon of New Caledonia, South Pacific. The lagoon is large and encompasses a highly diversified coral reef ecosystem where anthropogenic pressures are heterogeneously distributed, with various recreational uses such as fishing, boating, jet-ski and others. The lagoon comprises a network of MPAs including reefs and islets.

Monitoring biodiversity in this area is challenging due to its size, to species diversity, and to the variety of habitats, anthropogenic pressures and protection status encountered.

The observation design included two protected areas: Signal Islet and Larégnère Islet, where all fishing had been prohibited since 1989, and two adjacent unprotected reefs: Senez Reef and Larégnère Reef, as well as in the lagoon area between the two islets. Observations were conducted in the various habitats around these islets and reefs, including coral reef areas, seagrass beds and soft-bottom areas. Observations were realized at depths ranging between ∼1.5 m and 20 m.

### The STAVIRO observation system

**Figure 1:**
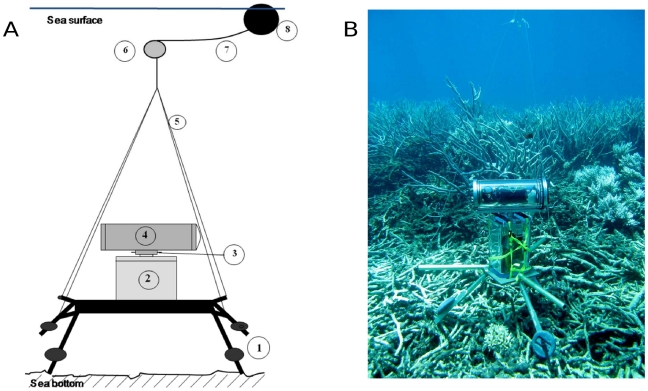
Description of the underwater rotating video system. (A) 1) weighted aluminium support; 2) engine housing; 3) rotating axis; 4) video camera housing; 5) nylon fishing line; 6) intermediate buoy; 7) floating rope; 8) surface buoy. (B) picture of the system in place.

The system consisted in two waterproof housings related by an axis. The lower housing contained an electric engine powered by 2.4 V rechargeable batteries which sets in motion the axis related to the upper housing enclosing the HD camera (Figure 1). The two housings were tied onto an aluminium support that was dropped from the boat onto the sea bottom. The support was rigged to an intermediate buoy that keeps the rigging tight, this buoy being itself fixed to a rope connected to a larger buoy in surface that was used to retrieve the system at the end of the observation.

The camera was a HD Sony™ camera HDR-SR11 with an integrated 30 Gigabyte hard drive enabling the recording of up to 4 hrs of HD images. The camera recorded a signal following the 1080i standard, i.e. with a full HD resolution of 1920×1080 pixels. Images were saved on the internal hard drive using the AVCHD™ format which is based on the MPEG-4 AVC/H.264 for image compression. The housing and camera resulted in an approximate focal angle of 60°.

The system is set on the sea floor and rotates at predefined time intervals from a fixed angle. Rotations of the housing camera were programmed via a timer enclosed in the engine housing.

### Observation protocol

After a number of trials, rotations were programmed so that the camera housing rotates from 60° every 30 seconds. Hence, six observation sectors were recorded per 360° rotation, and a rotation takes approximately three minutes. In order to gather information about the variability of fish presence and abundance, the system was left in place long enough to ensure at least three complete 360° rotations. Disturbances due to boat presence, engine noise and setting and retrieval of the system were minimized by leaving the system in place at least one minute before and after the three complete rotations. Overall, the system was left in place for around 12′. No artificial light was used to avoid disturbance and attraction.

Stations were regularly spaced within the study area using ArcGIS (ArcGIS (Version 9.3). ESRI. 2008) to ensure an appropriate spatial coverage. For stations located in rocky substrates, the distance between stations was approximately 200 m, while in soft bottom areas between the islets, stations were ca. 400 m apart. Slight deviations from this planned spacing incurred when the intended location was not fully suitable for setting the system horizontally.

Twenty-two day trips were organized between 18^th^ June and 31^th^ July 2007. Two systems were simultaneously used. After each day, the recorded video sequences were dumped from the camera hard drives on a PC hard drive. Each sequence was previewed and it was validated for analysis when i) at least three rotations could be analysed; and ii) horizontal underwater visibility was at least 5 m. When a station was not valid, it was carried out again the following day.

### Image analysis

Images were analysed using a standard viewing software that enabled slow view and zooming, such as PowerDVD (PowerDVD (Version 9.0 Ultra). Cyberlink Corp. 2009) or the Nero Suite (The Nero Suite (Version 9) Nero Ltd. 2009). Images were analysed per 60° observation sector. At all stations, individuals observed within a 5 m distance from the camera were identified and counted per species for a list comprising a large number of species, and commonly used in the UVC monitoring conducted in the region (Table 1). This list of 26 families comprises all fished species, Chaetodontidae, emblematic fish species, turtles, and Dugondidae. Species that live close to or in corals, such as Pomacentridae and Apogonidae, and small pelagic species, like Clupeidae and Engraulidae, were not counted in the present study, although they can be identified from images [23]. In many stations, horizontal underwater visibility was larger than 10 m. Individuals occurring at distances larger than 5 m were counted separately, and the observation distance was recorded, as is usually done during stationary UVC [24]. These individuals were analyzed separately.

**Table 1:** List of families identified and counted during image analysis.

Acanthuridae	Kyphosidae	Mullidae
Balistidae	Labridae:	Myliobatidae
Carangidae	*Bodianus*	Priacanthidae
Carcharhinidae	*Cheilinus*	Scaridae
Chaetodontidae	*Choerodon*	Scombridae
Chanidae	*Coris*	Serranidae
Dasyatidae	*Epibulus*	Siganidae
Ephippidae	*Hemigymnus*	Sphyraenidae
Haemulidae	*Oxycheilinus*	Zanclidae
Hemiramphidae	Lethrinidae	Cheloniidae
Holocentridae	Lutjanidae	Dugongidae

The list includes the fished species and emblematic species that can be observed visually (from UVC) or by video. For Labridae, only the genuses in italics were considered.

Images were analysed with the assistance of expert UVC divers, particularly at the onset of the analysis stage. Individuals were counted per sector, and then summed up for each 360° rotation. To minimize potential double counting, particular attention was given to the direction of fish movement with respect to camera rotation.

For each species and each station, we calculated the maximum abundance observed over the three rotations, and the mean abundance over those rotations, which averaged out the variability between rotations. Maximum abundance observed, also termed MaxN, is widely used for BRUV [25]. Abundances were expressed in densities, i.e. numbers of individuals per m^2^ (ind.m^−2^), which were computed from fish observed within a 5 m radius from the camera, based on the disk surface area. To assist in estimating whether the distance of fish to the camera was lesser or larger than 5 m, the person analyzing the images used screenshots of plastic fish silhouettes of several sizes (0.2 m, 0.4 m, 0.6 m, 0.8 m and 1 m) and colours (bright and dark ones), taken at several distances from the same camera (2 m, 5 m, 7 m and 10 m), following [26].

In addition to abundance estimation, fish size was estimated as small, medium or large, and corresponding size bounds were defined by expert UVC divers for each species.

Habitat was characterized using the medium-scale approach [27]. For each station, habitat was described by three sets of variables: i) substrate composition; ii) biotic cover; and iii) depth, bottom topography and complexity (Table 2). Substrate composition accounted for the granularity of abiotic bottom cover. Biotic cover included live coral, macroalgae and seagrass. Complexity quantified the number and variety of potential refuges. Habitat parameters were averaged over sectors at a given station, except for depth, which was recorded from the boat.

**Table 2:** Habitat description.

Parameter	Definition
Depth	Depth measured from boat when setting the system
Topography	If h denotes the largest altitude between troughs and elevations, values from 1 to 5 respectively correspond to h negligible, h<1 m, 1<h<2 m, 2<h<3 m, h>3 m
Complexity	Values from 1 to 5: none, low, medium, strong, outstanding
Substrate composition	Percent cover of mud, silt, sand, rubble, small boulder (<0.3 m), large boulder (0.3 m≪1 m), rock (>1 m), slab
Live coral	Percent cover of live coral
Macroalgae	Percent cover of macroalgae
Seagrass	Percent cover of seagrass

### Data analysis

Habitat data were analysed in two ways. Firstly, the distributions of percent covers of live coral and seagrass were modelled as a function of protection status using binomial GLM, and the effects of the two factors were statistically tested. Secondly, habitat data were used to construct a typology of stations based on the main habitats encountered in the studied area. In order to account for the three groups of variables describing habitat, the typology was achieved in several steps. A Principal Component Analysis (PCA) was first carried out on substrate composition data, followed by an Hierarchical Ascending Cluster Analysis (HACA) based on Ward's distance [28]. The resulting cluster index was then considered along with the other variables describing habitat (Table 2) in a Multiple Correspondence Analysis followed by an HACA. For this purpose, depth was coded into four categories 0 to 5 m, 5 to 10 m, 10 to 15 m, and 15 to 20 m. Topography and complexity were coded into three categories (1, 1 to 2.5, and 2.5 to 5) based on the distribution of average values per station, while biotic covers of coral, seagrass and macroalgae were coded into four categories using 33% and 66% percentiles as intermediate bounds. The clusters of stations resulting from the typology were subsequently described using the initial habitat variables, by testing the difference in the frequency of each category of a given variable between the cluster and the whole set of stations. Such differences were statistically tested using t-statistics based on a Gaussian assumption [29]. A significant test indicated that the frequency of the category was higher (positive statistic) or lower (negative statistic) in the cluster compared with the whole set of stations. In this case, the category was considered to significantly explain the cluster. The resulting typology assigned a habitat cluster index to each station, which was subsequently used as a habitat factor when analyzing spatial variations in vagile macrofauna.

With respect to the latter, we first investigated the frequency and abundance density of each taxonomic family in the data set. Observed frequencies were qualitatively compared with published studies involving UVC, and conducted in the same area and habitats ([30], [31], [32]). [30], [32] were conducted on the barrier reef in the same lagoon area in 1993, 1995 (and 2001 for [32]), while [31] encompassed five islets in the area, including the site studied in the present paper, and data were collected in 1985, 1990 and 1994.

Secondly, the overall density and species richness per station were computed as synthetic metrics of biodiversity. In a third step, we focused on the abundance of three key fished species and on the abundance of Chaetodontidae. Finally, we analyzed the frequency of emblematic species of the lagoon, such as turtles, sharks, rays and humphead wrasse (*Cheilinus undulatus*).

Each of the above metrics was modeled with respect to protection status and habitat using a two-way General Linear Model. For each metric (except for frequencies), the appropriate statistical distribution was selected as the one minimizing Akaike's criterion ([33]) among the Gaussian, log-normal, Poisson and negative binomial distributions. The frequency of emblematic species was modelled through presence/absence data using a binomial GLM model to test the effects of protection status and habitat. Spatial differences in species richness, abundance density and presence/absence data were then tested from multiple comparisons [34].

## Results

### Implementation of the technique

The observations were collected over twenty-two days of field work, by two persons in addition to the pilot, and in some days, only one person with the help of the pilot for hauling the system aboard.

In very shallow waters (less than 1.4 m), it was more appropriate to leave the system without the rigging so that it did not entangle in the camera housing when rotating. As the system was still under validation, no station was set at depths larger than 20 m, but the housings could actually endure larger depths.

Out of the 317 stations realized, 221 (i.e. 70%) of the stations, were validated for image analysis. Non-exploitable stations resulted from defects in rotation, recording or camera autofocus, and sometimes the system fell or was not horizontal. In few cases, only one system was used due to technical problems. On average, 14.4 stations were obtained in a given day, corresponding to 3.7 stations per hour, with a mean observation time per day of ca. four hours. The maximum number of stations obtained during a single day was 31. Station depth ranged between 1.2 and 20 m.

### Image analysis

The analysis time per station ranged between 10 minutes and 1 hour and 15 minutes for fish, and ca. 5 minutes for habitat.

Images were analysed for 196 stations out of the 221 valid ones. The rest of the stations were close to other stations and the underwater visibility was less good than at neighboring stations.

Among the 196 stations analysed, no vagile macrofauna was seen in 58 stations. In the 138 other stations, 10357 individuals were counted, corresponding to 149 species and 23 families (Table 3). 148 (i.e. 1.4%) individuals could only be identified at the family level, while 560 (i.e. 5.4%) were identified only at the genus level. These were mainly Lethrinidae (328 ind.), *Scarus* (228 ind.), *Acanthurus* (92 ind.). Overall, 6.8% of individuals were not identified at the species level, in general because they were too far or swam through the field of vision too quickly.

**Table 3:** Species number, abundance and frequency of the families observed in the 196 stations.

	2007 STAVIRO		[35]	[31]	[32]
Nb. observations	196	196	157	132	212
Numbers observed	Individuals	Species	Species	Species	Species
Chaetodontidae	227	20	22	31	Na
Acanthuridae	1444	17	23	24	25
Mullidae	805	12	12	14	13
Scaridae	873	12	24	22	22
Serranidae	179	12	23	25	21
Labridae	301	11	12	10	17
Lutjanidae	3591	11	11	18	13
Lethrinidae	1492	10	17	18	17
Carangidae	173	8	5	11	7
Balistidae	262	7	12	0	6
Siganidae	462	6	7	9	8
Haemulidae	62	4	4	9	5
Carcharhinidae	14	3	3	Na	3
Cheloniidae	13	3	Na	Na	Na
Scombridae	47	3	1	1	1
Dasyatidae	9	2	2	0	2
Kyphosidae	225	2	1	1	1
Chanidae	72	1	Na	1	Na
Ephippidae	44	1	0	0	1
Holocentridae	1	1	1	Na	6
Myliobatidae	3	1	1	0	1
Priacanthidae	39	1	0	1	0
Zanclidae	19	1	1	Na	1

The last three columns contain the number of species per family usually observed in UVC transects realized in the same habitats in the same area [31], [32], [35]. The number of Labridae species corresponds to the genus listed in Table 1. The number of Holocentridae species corresponds to the only fished species in the family. Na means the family was not observed in the cited reference.

### Biotic cover and habitat

The observations encompassed various habitats. Live coral cover was zero in 50.5% of observations, and it was larger than 33% at 7% of observations. Seagrass and macroalgae were absent from 49% and 25% of stations, respectively.

**Figure 2:**
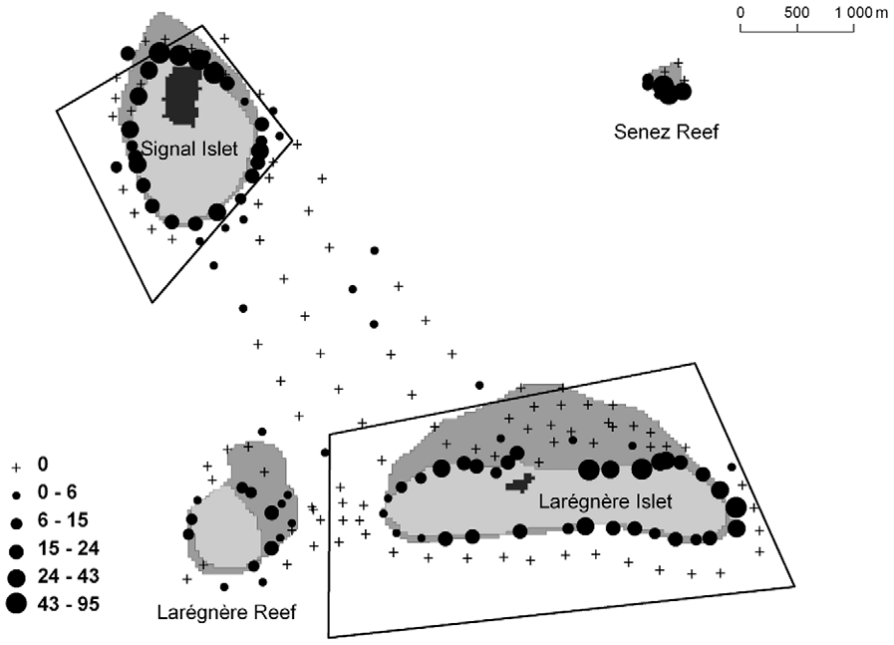
Map of live coral percent cover. Circles are proportional to percent cover (see legend in insert).

**Figure 3:**
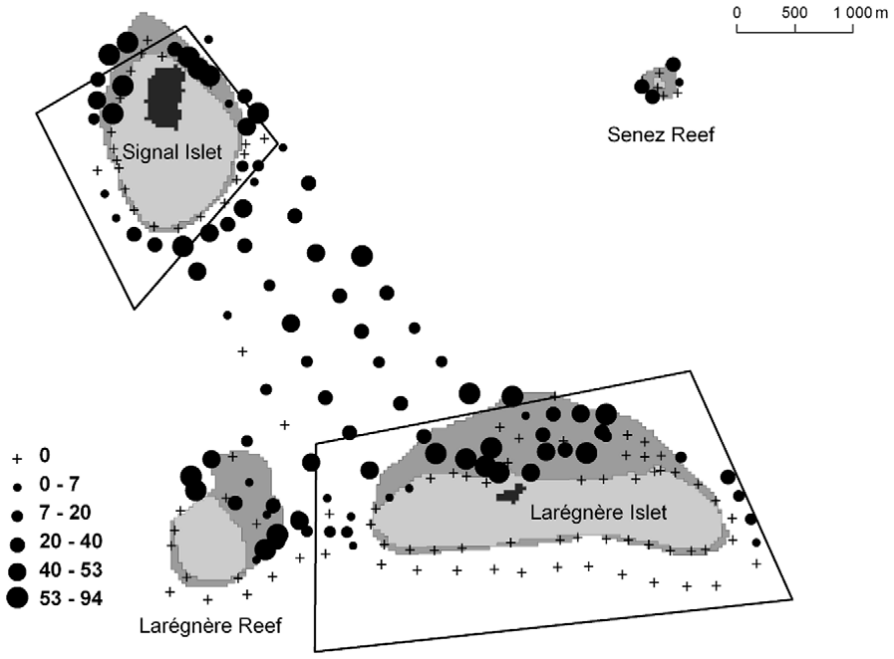
Map of seagrass percent cover. Circles are proportional to percent cover (see legend in insert).

**Figure 4:**
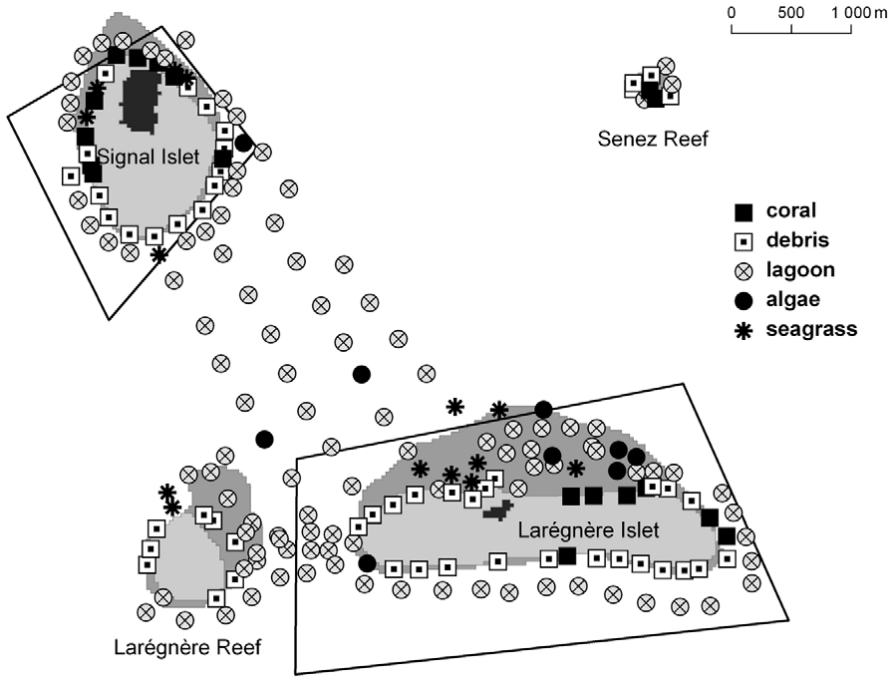
Distribution of habitats resulting from the typology of stations (see legend in insert for symbol definition). Clusters were described in Table 4.

Live coral could be observed on the reef slope around the two islets and the two reefs (Figure 2). It was larger on the leeward side of the islets. Data also showed the presence of live coral patches between the islets and in a few stations further from the islets and reefs. Seagrass was absent from the windward side of Larégnère Islet and Larégnère Reef which were less sheltered than the windward side of Signal Islet (Figure 3). In reverse, it was found in most stations on the leeward sides of the islets and reefs, and in the lagoon area between the two islets. At the eastern tip of Larégnère Islet, seagrass was absent but macroalgae were abundant (see typology on Figure 4).

The typology led to a partition of the stations into 5 clearly separated clusters (Table 4). The larger cluster (lagoon, 105 stations) included stations with an average 87.5% sand cover, depths larger than 10 m at two thirds of the stations. Live coral was absent from 78% of these stations, and seagrass and macroalgae cover were lower than on average over all stations. The second largest cluster (50 stations) mostly comprised stations with a substrate of mixed debris consisting of rubbles (average 30.1%), sand (average 38.6%) and hard coral (average 21.7%), and corresponding to intermediate depths (Table 4). Seagrass and macroalgae were absent from 82% and 60% of the stations respectively. Stations with a large live coral cover constituted a cluster of 18 stations. Coral cover was high (average 46.8%), and seagrass was absent from all stations. Substrate was mixed and rocky, including hard coral cover (average 61.8%), slab (average 9.0%), and stations were shallow (average depth 4.4 m). Complexity was higher than in the rest of the stations. The seagrass cluster comprised 14 stations with a seagrass cover larger than 66% on a sandy substrate, and with a medium complexity. The smallest cluster (9 stations) gathered all stations where macroalgae cover was larger than 66%. Seagrass was absent from 89% of these stations. In clusters 1, 2 and 3, sand cover was higher than 87% on average, while it was clearly lesser in clusters 4 (average 38.6%) and 5 (average 12.4%) (Table 4). The spatial distribution of clusters confirmed that the living coral habitat was located on the leeward side of the islets, while debris dominated stations were found on their windward side (Figure 4). Stations from the largest cluster were situated in the lagoon area between the islets and around the islets at a small distance. Seagrass was mainly encountered close to the islets on the sheltered side.

**Table 4:** Description of the habitat clusters resulting from the typology.

	Explanatory variable						
Cluster	Substrate	Depth (m)	Topo graphy	Comple xity	Live coral (%)	Seagrass (%)	Macroalgae (%)
Lagoon (105)	sand *(p<6.10^−25^)*	**11.0** *(p<7.10^−4^)*	1.5 *(p<2.10^−6^)*	1.9 *(p<3.10^−6^)*	0.8 *(p<4.10^−17^)*	**20.1** *(p<3.10^−6^)*	**24.3** *(p<2.5.10^−10^)*
Sea grass (14)	sand *(p<5.10^−3^)*	7.5 *(NS)*	1.5 *(NS)*	2.0 *(p<5.10^−3^)*	0.9 *(NS)*	**80.0** *(p<2.2.10^−21^)*	3.0 *(NS)*
Macro algae (9)	sand	7.6 *(NS)*	1.9 *(NS)*	**2.5** *(NS)*	0.5 *(NS)*	1.6 *(p<3.10^−2^)*	**82.6** *(p<2.10^−15^)*
Debris (50)	mixed sand and rocky *(p<2.3.10^−19^)*	**6.7** *(p<7.10^−6^)*	**2.2** *(p<3.10^−2^)*	**2.4** *(p<3.10^−3^)*	**16.0** *(p<9.10^−22^)*	4.0 *(p<7.10^−8^)*	2.5 *(p<7.10^−10^)*
Coral (18)	mixed rocky *(p<5.10^−21^)*	**4.4** *(p<5.10^−3^)*	**2.6** *(p<2.10^−10^)*	**3.3** *(p<6.10^−12^)*	**46.8** *(p<8.10^−15^)*	**0.0** *(p<2.10^−6^)*	0.3 *(p<3.10^−5^)*
All stations (196)	–	**8.9**	**1.8**	**2.2**	**8.9**	**17.6**	**17.7**

The number of stations belonging to each cluster was reported between parentheses. For a given explanatory variable, the number of stations displaying the characteristic was reported, along with the p-value corresponding to the test of the difference in proportion between the cluster and the whole set of 196 stations.

### Species and abundances observed

149 species belonging to 23 families were observed among the list of 26 families (Tables 3 versus Table 1). In order to provide qualitative elements of comparison, we reported the number of species observed from UVC transects conducted with the same or a very similar list of censused species, in the same panel of habitats within the same lagoon area [31], [32], [35] (Table 3). In each reference cited, a transect corresponded to an approximate surveyed surface of 200 m^2^. The area analysed in video stations was limited to ca. 78.5 m2 for density estimates and species richness of fish species (but rays and sharks), because of the observation radius of 5 m (see § 2.4), but it rose up to ∼300 m^2^ for occurrence counts of large emblematic species (sharks, rays and turtles) when visibility was high. On average, 82% of the species observed in these UVC were also detected from our stations.

**Figure 5:**
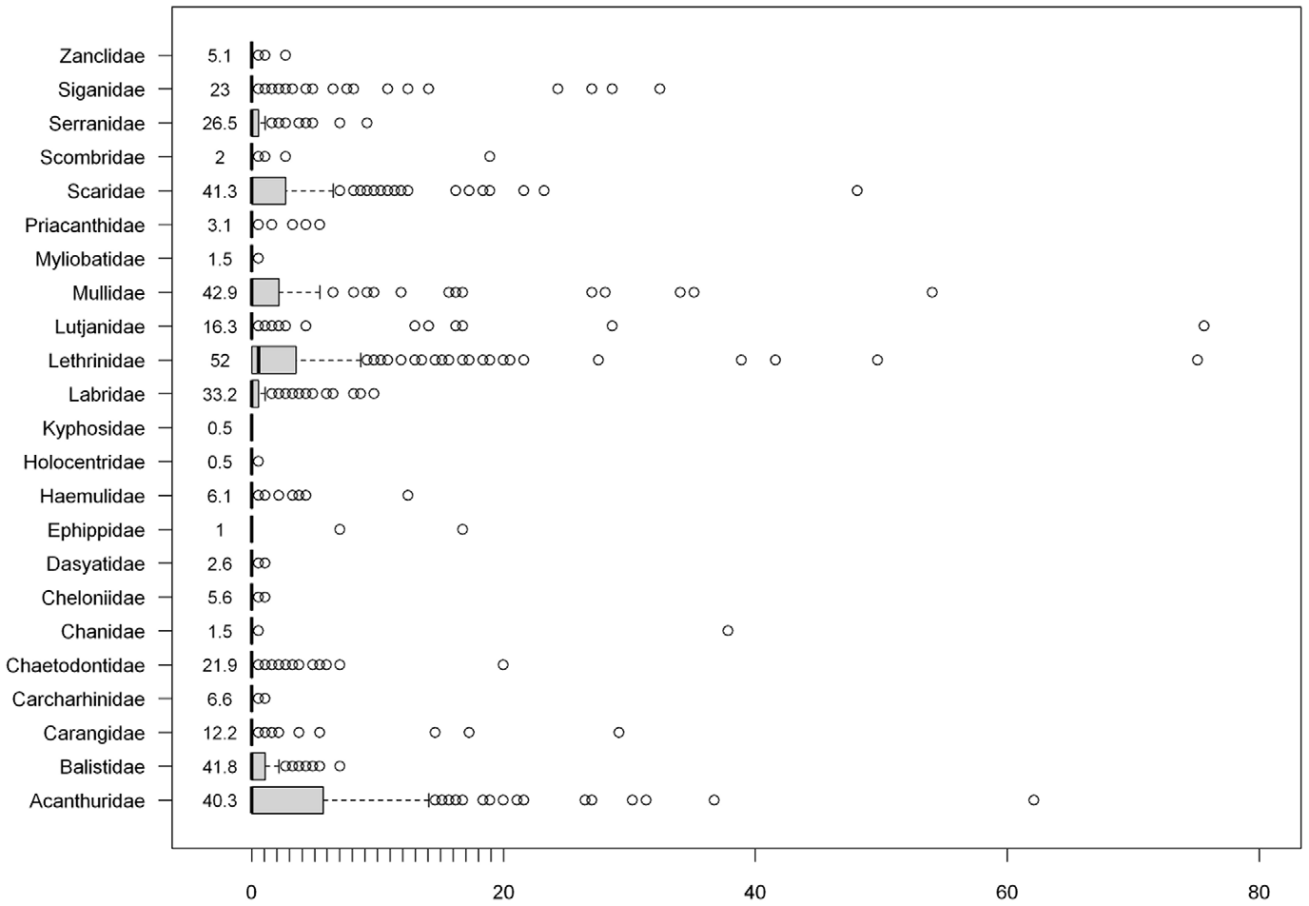
Distribution of abundance density per family. Abundance density was the average over rotations. On each boxplot, boxes showed the interquartile (0.25, 0.75) ranges; whiskers extended to the data point at ≤1.5 times the box length away from the box; values outside this range were represented by dots. Four outlying values were not reported for better readability of the plot : 810.6 ind/100 m^2^ (two values) and 129.7 ind/100 m^2^ for Lutjanidae, and 95.5 ind/100 m^2^ for Kyphosidae. The frequency (in % stations where the family was seen) was reported on the left of the boxplots for each family.

In the rotating video, the most often observed families were Chaetodontidae, Acanthuridae, Scaridae, Serranidae, Lutjanidae, Mullidae, Labridae and Lethrinidae, which also were the most common families observed in UVC (see frequencies per family on Figure 5). Lethrinidae were seen in 52% of the stations, while Mullidae, Scaridae, Acanthuridae and Balistidae were seen in ca. 40% of the stations. Labridae were observed in one third of the stations, and Siganidae, Serranidae, Chaetodontidae were observed in one fourth of the stations.

Maximum abundance densities were observed for Lutjanidae (8.11 ind.m^−2^ and 1.30 ind.m^−2^) (mostly due to fish schools of *Lutjanus quinquelineatus* and *L. fulviflamma*), Kyphosidae (0.96 ind.m^−2^). Lethrinidae and Acanthuridae were frequently observed in large abundances, and to a lesser extent Scaridae and Mullidae (Figure 5).

**Figure 6:**
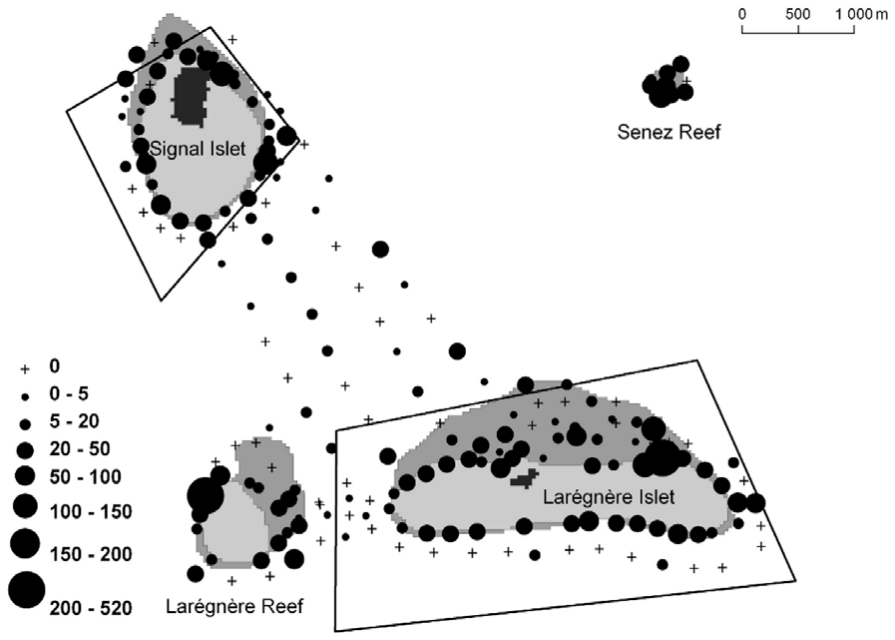
Maximum overall abundance per station. Circles are proportional to the maximum observed abundance density per station (i.e. MaxN) (see legend in insert, densities were plotted in #ind/100 m^2^).

Overall abundance density per station was larger on the reef slopes of the islets and reefs (Figure 6). But non-negligible densities were also observed in the lagoon area between the two islets. Mean and maximum overall densities per station significantly differed according to habitat (GLM with Gamma distribution, p<2.10^−16^ in both cases), but not according to protection status.

**Figure 7:**
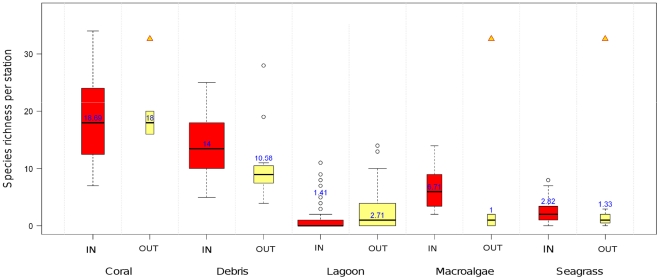
Species richness per station as a function of habitat (see **Table 4** for habitat definition) and protection status (IN versus OUT). On each boxplot, boxes showed the interquartile (0.25, 0.75) ranges; whiskers extended to the data point at ≤1.5 times the box length away from the box; values outside this range were represented by dots. Triangles indicated boxplots with less than 5 data.

Species richness was found to strongly depend upon habitat (Figure 7). Using a GLM model with a Gamma distribution, the effect of protection status was not significant, but the habitat effect was clear (p<2.10^−16^) and interactions were significant (p<0.04). Multiple comparisons evidenced that species richness was significantly higher in coral, debris and macroalgae habitats compared to the lagoon and seagrass ones (p<0.001). In every habitat, species richness did not significantly differ between protected and unprotected areas.

**Figure 8:**
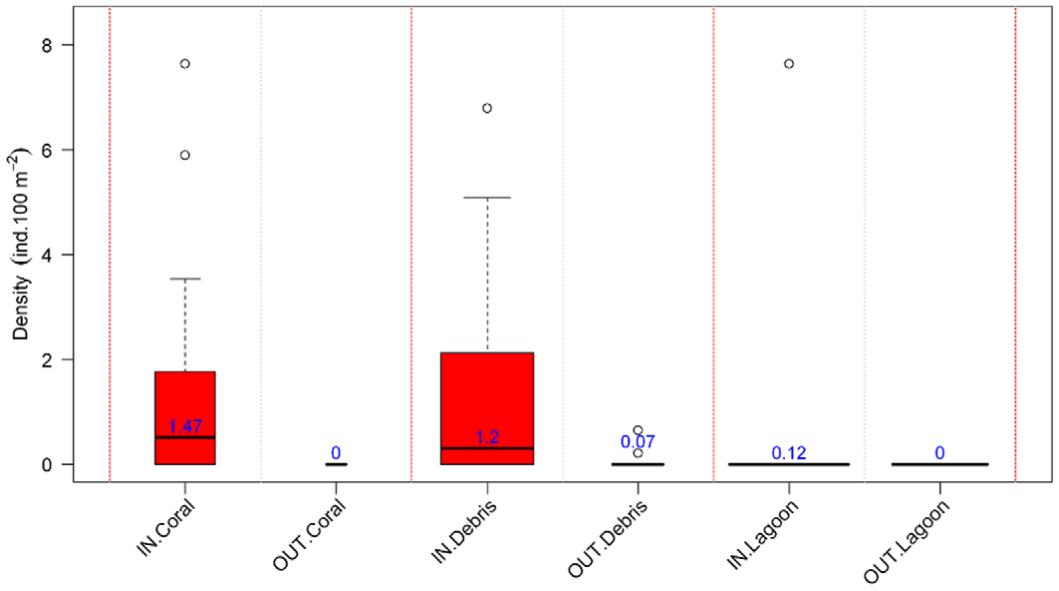
Distribution of *Naso unicornis* abundance density according to habitat (lagoon, debris and coral) and protection status (IN versus OUT of protected area). The species was not found in the other two habitats. Abundance density was the average over rotations. On each boxplot, boxes showed the interquartile (0.25, 0.75) ranges; whiskers extended to the data point at ≤1.5 times the box length away from the box; values outside this range were represented by dots. Triangles indicated boxplots with less than 5 data.

The abundance densities of three major fish species were then investigated. The leopard coralgrouper *Plectropomus leopardus*, a favorite target species for spearfishers, was observed in the three habitats lagoon, coral, and, in smaller densities, on debris. Density per station differed according to habitat (GLM with Gamma distribution, p<2.4.10^−5^), but not according to protection status. The spangled emperor *Lethrinus nebulosus*, a target species of line fishing was mainly observed in the seagrass habitat, and to a lesser extent in the lagoon habitat. Density per station significantly differed according to habitat (GLM with Gamma distribution, p<0.0032), but not according to protection status. The third target species, the bluespin unicornfish *Naso unicornis*, was another important target of spearfishing (Figure 8). The density of *N. unicornis* was also modeled from a GLM with a Gamma distribution considering the two factors habitat and protection status. Main effects and interactions between the two factors were significant (habitat: p<0.0022, protection status: p<0.031, and interactions: p<0.0087). The density of the species was thus larger in the protected areas, and particularly so in the coral and debris habitats which were preferred by the species.

In the next step, we considered the density of Chaetodontidae, which are indicators of coral health status and abundance (see e.g. [36]). The family was absent from the seagrass habitat. A GLM with a Gamma distribution fitted to the density of Chaetodontidae on the other four habitats showed a significant habitat effect (p<10^−6^), but no effect of protection status. Multiple comparisons evidenced significantly higher densities in the coral and debris habitats than in the lagoon habitat (respectively 2.9 ind/100 m^2^ and 0.67 ind/100 m^2^, versus 0.11 ind/100 m^2^). The relationship between Chaetodontidae density and live coral cover was thus clearly detected from the data.

**Figure 9:**
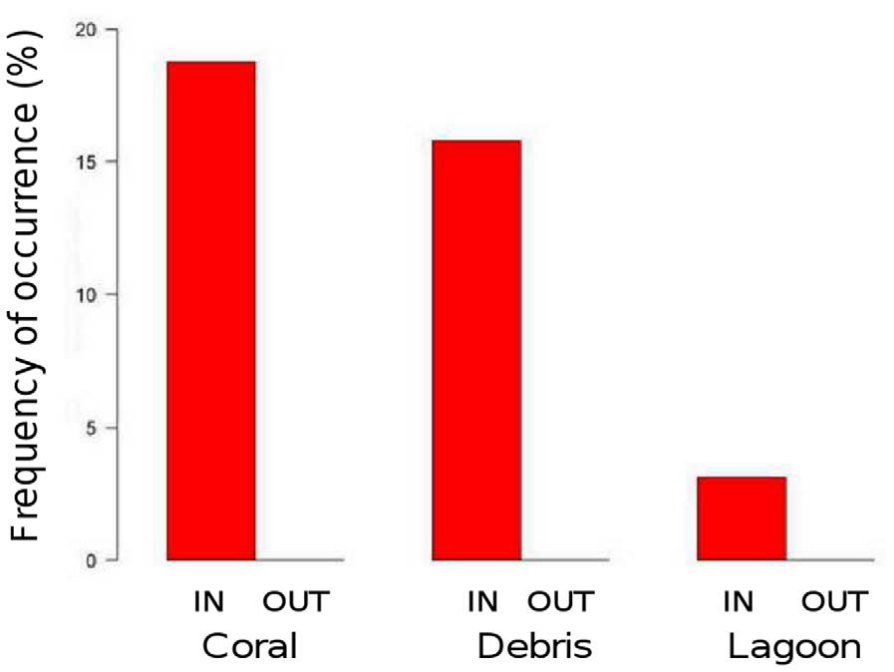
Frequency of occurrence of marine turtles. Frequencies were plotted per habitat (labeled on the X-axis) and per protection status (protected areas in dark grey and unprotected areas in light grey).

**Figure 10:**
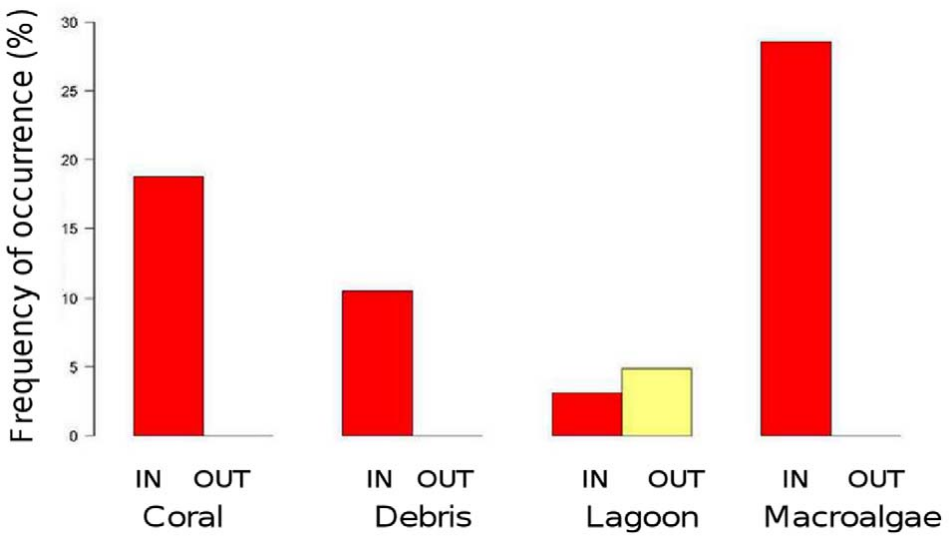
Frequency of occurrence of sharks. Frequencies were plotted per habitat (labeled on the X-axis) and per protection status (protected areas in dark grey and unprotected areas in light grey).

Emblematic and rare species were the final focus of the study. The frequency of occurrence of marine turtles (Chelonidae), sharks (Carcharinidae), rays (Dasyatidae and Myliobatidae) and humphead wrasse (*Cheilinus undulatus*) were computed per habitat and protection status. Three turtle species were encountered in three habitats (coral, debris and lagoon), in relation with their preferenda, but also because these habitats comprised more stations than the other ones (Figure 9). Turtles were systematically observed in protected areas. From a binomial GLM model of presence/absence restricted to the coral, debris and lagoon habitats, both habitat and protection status effects were found to be significant (p<0.01 for both effects). Sharks were observed in four habitats, mostly in protected areas (Figure 10). Rays were observed in seven stations mostly in the lagoon habitat and once in the macroalgae habitat (results not reported). *Cheilinus undulatus* was encountered in the coral and debris habitats, only in protected areas (results not reported). Presence/absence GLM modeling did not evidence any significant effect of habitat or protection status for none of these three species groups. Rare species such as turtles, sharks, and rays could thus be observed at a non-negligible number of stations (Table 5).

**Table 5:** Frequency of occurrence of emblematic species in the video stations.

Emblematic species	Overall frequency in the 196 stations	Highest frequencies in given habitats
Marine turtles	6.6%	19% in protected coral habitat (16 stations) 16% in protected debris habitat (38 stations)
Sharks	7%	28.5% in protected algae habitat (7 stations)
Rays	6.1%	50% in protected algae habitat (2 stations) 6.7% in lagoon habitat (105 stations)
Humphead wrasse	1.0%	6.3% in protected coral habitat (16 stations)

## Discussion

### Observations of biodiversity

In the present study, 149 species from 23 taxonomic families were observed.

A quantitative comparison of UVC transects and HD video transects conducted with the same type of camera also showed that HD video detected reasonably well the species and individuals observed in UVC [23], since 85% of species seen by UVC were also detected by HD video (based on the analysis of all fish species, unlike the present analysis which focused on a list of species, see Table 1). In the latter paper and in the present study, the proportion of species that were not identified up to the species level remained very low. Large abundances of fish species could be observed from the HD rotating video, in particular fished species. Carangidae, Carcharinidae, Dasyatidae, Myliobatidae and Chelonidae were observed at a number of stations. These outcomes were likely explained by i) the absence of a diver, ii) by the larger variety of habitats investigated using this technique, including seagrass and other soft bottoms; and iii) by the large number of stations realized.

Video observations of macrofauna compared in a satisfactory way with those from UVC surveys conducted in the same area and in the same habitats, although quantitative comparisons could not be conducted as the timings of the studies, the number of observations and the observation surface area were very different. Note that the objective of this study being to demonstrate the adequacy of the technique for spatial survey, no formal comparison with UVC was carried out here.

Our results showed that the technique detected well the species and families observed in UVC, even in this highly diversified coral reef ecosystem. A pilot study was conducted in 2010 in rocky and seagrass habitats in a Mediterranean ecosystem, during which 33 species could be observed from 22 STAVIRO stations (Pelletier and Hervé, unpubl. data).

In addition, video provided information about habitat and particularly biotic cover at no extra cost along with fish data. Analysing images for abiotic substrate and biotic cover proved to be easy and quick. Simultaneous information on vagile macrofauna and habitat collected at a large number of stations are a valuable asset for analyzing spatial and temporal patterns of macrofauna in relation with both habitat and anthropogenic pressures.

### Implementation of the technique

As far as field work is concerned, 70% of the stations were validated during the 2007 survey, which was high for a first test of the technique in the field. The few technical problems encountered in 2007 were easily solved, since 1246 validated stations (corresponding to a 81% validation rate) could be realized in 2008, 2009 and 2010 in the same area and in similar places in the New Caledonian lagoon (results not reported here, Pelletier and Mallet, unpubl. data). Defective stations only resulted from a poor underwater visibility in relation with weather conditions. In these surveys, the mean number of observations per day obtained from two rotating systems was 30 stations for ca. 6 hrs of field work. Note that as the technique is easy to implement and requires limited logistics, it is possible to target favorable conditions for field work.

Overall, the system proved to be remarkably stable and was used in depths ranging from 1.2 m to 27 m, and in various sea and weather conditions. The number of observations that can be realized in a given period of time depends on the travel distance between two stations; it is thus recommended to realize pairs of stations that are not too far apart.

The main advantages of the technique presented in this paper lie in the fact that i) a large number of observations can be realized on the field within a short period of time; ii) it can be implemented easily on site by technical staff following a repeatable protocol. This enables to cover large areas including many habitats, and to obtain comparable data from several sites. Another major advantage is to avoid on-site observer effects. As for drawbacks, the system needs to be set horizontally and on a steady bottom like most remote systems. Setting the system on outer slopes or in areas fully covered with live coral may thus take a few minutes.

At the laboratory, images were analyzed by two persons, particularly at the onset, to build the capacity for identifying species and counting individuals from images. Analyses for fish assemblages were carried out with the help of an experienced UVC fish expert. Image analysis was greatly enhanced by the use of HD as was also found by [37]. Between 2007 and 2010, several persons were trained to image analysis, and this experience showed that the continuous presence of a fish expert was not needed. Because images can be viewed as many times as needed, image analysis can proceed in a flexible way, using identification guides and building on previous analyses. Training a person to analysis required at most one month.

### Cost-effectiveness of observation techniques

Three techniques were quoted in this article: STAVIRO, UVC and BRUV. We herebelow provided a tentative comparison of UVC and STAVIRO in the light of spatial survey of biodiversity (Table 6). BRUV were not included in Table 6 as field work parameters for the technique were not available in the study area. From the literature, BRUV shares with STAVIRO the advantages inherent to the absence of a diver. The use of a bait permits to increase the number of individuals observed by attraction of carnivorous (and other) species, but the uncertainty about the bait plume hampers the estimation of density estimates. Baiting also requires that the station be left at least 35′ to 60′ in place to ensure bait effectiveness [38].

**Table 6:** Cost-effectiveness of UVC and STAVIRO techniques.

Observation technique	Time (observation, image analysis data input)	Staff required Nb of observations per day	Advantage
UVC	• On site: 45′ to 90′ per fish transect & 10′ per habitat transect• At the office :15′ for data input	• 2 divers (1 fish expert, 1 for habitat)• Max. 3 transects per day and per diver	• Widely used• Mostly field work• Little work after data collection
STAVIRO	• On site: 15′ per station• At the office: 10′ to 65′ per station for fish et 10′ for habitat (image analysis+data input)	• On site: 2 technical staff• 20 stations per day with 1 system, 30 stations per day with 2 systems• At the office: 1 trained person(1), possible validation by fish expert	• Spatial coverage• No diver effect on fish nor on data collection• Simultaneous data on macrofauna and habitat• Single protocol• Reduced time at sea• Larger range of depths• Archiving of data• Several analyses possible

(1) Training period, one month maximum.

The STAVIRO does not rely on a diver or on a bait. Although it cannot be ascertained that the presence of the system underwater does not influence the vagile macrofauna, STAVIRO is certainly less obtrusive than UVC and BRUV.

Unlike UVC, many STAVIRO stations can be realized within a given amount of time. The time spared at sea for each station is utilized at the office to analyse images, and the overall time needed per observation is approximately the same for UVC and STAVIRO (Table 6). But field work can be realized by non-expert staff.

In developing the STAVIRO technique, priority was given to facilitate and speed up implementation in the field, while keeping the system as affordable as possible in order to foster its use. Hence, size estimation was not central, and abundances per size class were deemed precise enough. For the same reason, the system did not include a device for estimating the distance to the fish on the image. Similar to UVC training, observers were trained to estimate distances from underwater images displaying fish silhouettes from different sizes placed at several known distances (Mallet, unpubl. data).

Archiving data is a very important point with video observation. Although filing HD images raises issues of storage, archiving bears several advantages. Firstly, information is fully traceable, which is desirable for monitoring and reporting. Secondly, several independent analyses can be carried out, either for different needs corresponding e.g. to distinct levels of analysis, or to check previous analyses. Thirdly, images may be very useful for communication purposes, as they speak for themselves. This might be invaluable for reporting monitoring results, and also for educational purposes.

HD rotating videos thus appear as a promising observation technique for investigating spatial distributions of biodiversity and their evolution over time. Its advantages make it interesting for research and for monitoring the performance of conservation strategies such as MPAs. The implementation of a monitoring network of STAVIRO to monitor biodiversity in the New Caledonian lagoon is presently under development. This network would support reporting on biodiversity status for World Heritage sites (http://whc.unesco.org/en/). As the technique can be implemented by non-expert staff, it could be relevant in participatory community-based management involving local stakeholders, an approach that is being advocated for an improved governance of coastal areas [39], [40].

Being suited for both research and monitoring, this technique might support adaptive management of marine ecosystems [41], in that properly designed observation networks could at the same time inform the monitoring process and provide valuable information for research about biodiversity restoration in protected areas, and more generally about ecosystem resilience. Given the expected trends in marine biodiversity and the global conservation commitments, proactive management strategies would certainly benefit from the development and wide use of such tools.
